# Genetic diversity and shallow genetic differentiation of the endangered scaly‐sided merganser *Mergus squamatus*

**DOI:** 10.1002/ece3.70011

**Published:** 2024-07-09

**Authors:** Yulong Shen, Ye Gong, Diana Solovyeva, Lin Wang, Mu Li, Mengxuan Hu, Yiwei Jiang, Sergey Vartanyan, Haitao Wang

**Affiliations:** ^1^ School of Life Sciences Northeast Normal University Changchun China; ^2^ Institute of Biological Problems of the North FEB RAS Magadan Russia; ^3^ Northeast Institute of Geography and Agroecology, Chinese Academy of Sciences Changchun China; ^4^ North‐East Interdisciplinary Scientific Research Institute n. a. N. A. Shilo, Far East Branch, Russian Academy of Sciences Magadan Russia

**Keywords:** conservation, genetic diversity, *Mergus squamatus*, microsatellites, mtDNA, population structure

## Abstract

Examining patterns of genetic diversity are crucial for conservation planning on endangered species, while inferring the underlying process of recent anthropogenic habitat modifications in the context potential long‐term demographic changes remains challenging. The globally endangered scaly‐sided merganser (SSME), *Mergus squamatus*, is endemic to a narrow range in Northeast Asia, and its population has recently been contracted into two main breeding areas. Although low genetic diversity has been suggested in the Russian population, the genetic status and demographic history of these individuals have not been fully elucidated. We therefore examined the genetic diversity and structure of the breeding populations of the SSME and investigated the relative importance of historical and recent demographic changes to the present‐day pattern of genetic diversity. Using 10 nuclear microsatellite (SSR) markers and mitochondrial DNA (mtDNA) control region sequences, we found limited female‐inherited genetic diversity and a high level of nuclear genetic diversity. In addition, analysis of both markers consistently revealed significant but weak divergence between the breeding populations. Inconsistent demographic history parameters calculated from mtDNA and bottleneck analysis results based on SSR suggested a stable historical effective population size. By applying approximate Bayesian computation, it was estimated that populations started to genetically diverge from each other due to recent fragmentation events caused by anthropogenic effects rather than isolation during Last Glacial Maximum (LGM) and post‐LGM recolonization. These results suggest that limited historical population size and shallow evolutionary history may be potential factors contributing to the contemporary genetic diversity pattern of breeding SSME populations. Conservation efforts should focus on protecting the current breeding habitats from further destruction, with priority given to both the Russian and Chinese population, as well as restoring the connected suitable breeding grounds.

## Graphical abstract

Using mitochondrial DNA (mtDNA) control region sequences and nuclear microsatellite (SSR) markers, we found limited female‐inherited genetic diversity and a high level of nuclear genetic diversity. In addition, analysis of both markers consistently revealed significant but weak divergence between the breeding populations. Inconsistent demographic history parameters calculated from mtDNA and bottleneck analysis results based on SSR suggested a stable historical effective population size. By applying approximate Bayesian computation, it was estimated that populations started to genetically diverge from each other due to recent fragmentation events caused by anthropogenic effects rather than isolation during Last Glacial Maximum (LGM) and post‐LGM recolonization.

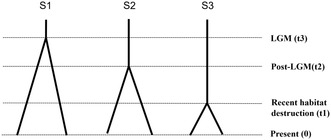

## 1 INTRODUCTION

Endangered species often have small populations, resulting in adverse effects on fitness due to reduced genetic variability; thus, they have greater extinction risks than their common counterparts (Frankham, 2002; Wilder et al., 2023). When their distribution ranges are increasingly restricted and isolated, species' extinction risks may increase through demographic and genetic stochasticity of the small populations (Lande, 1998). Over the past century, anthropogenic habitat modifications have made natural habitats increasingly small and fragmented for many endangered species (IUCN, 2023), likely resulting in detrimental impacts on the genetic diversity of small populations (Kleinhans & Willows‐Munro, 2019; Lindsay et al., 2008; Palomares et al., 2012). However, past drastic demographic changes during naturally occurring historic fragmentation events (such as isolation during glaciation and heterogeneous postglacial recolonization; Jones et al., 2005; Lait et al., 2012) may confuse the effects derived from recent anthropogenic changes (Weinberger et al., 2021). In addition, relative role of modern and historical factors could be different among different species (Matocq & Villablanca, 2001; Tracy & Jamieson, 2011; Wang et al., 2019). It is thus challenging to distinguish recent factors from historical effects on shaping current genetic diversity patterns in many endangered species. To inform conservation planning (e.g., defining conservation units and networks of protection areas; McInerney et al., 2012; Moritz, 1994) in endangered species, genetic variability, population structure (Lande, 1988; Lettink et al., 2002), and historical processes that produce and sustain the genetic patterns are essential (Moritz, 2002).

The globally endangered scaly‐sided merganser (SSME; *Mergus squamatus*) is a riverine specialist (Buckton & Ormerod, 2002) breeding in a narrow range of Northeast Asia, and wintering in central and southern China (Zeng et al., 2015, 2018). Suitable breeding habitats and submontane stream forests with unique physical environmental characteristics (Xu, Wang, et al., 2021a) have been severely disrupted by logging, hunting, and other human disturbances (IUCN, 2017). Historically, the breeding populations have been restricted mainly to temperate mountainous riparian old‐growth forests in Southeast Russia and Northeast China (IUCN, 2017). However, coinciding with economic development since the last century, the SSME has become less common in many of its previously established habitats (BirdLife International, 2001; Solovyeva et al., 2014). Extensive field surveys and breeding habitat modeling have suggested that the global breeding range of SSME has recently contracted into two main areas, that is, the Sikhote‐Alin Range of Russia and the Changbai Mountains of China (Xu, Solovyeva, et al., 2021b). The Sikhote‐Alin population is estimated to include 1643 pairs after an abrupt decline during the 1960s–1970s (Solovyeva et al., 2014). However, only 166 breeding pairs remain in the Changbai Mountains of China, and the small population is suspected to undergo a continuing and rapid decline as a result of habitat destruction, illegal hunting, and disturbance (Liu et al., 2010).

The restricted distribution and recent extreme habitat loss may have caused genetic bottlenecks and genetic differentiation, resulting in decreased genetic diversity, such as that found in its counterpart in the Neotropical realm, the Brazilian Merganser *Mergus octosetaceus* (Maia et al., 2020). The SSME was found to exhibit lower levels of mtDNA genetic diversity than some other closely related sea ducks and other bird species (Solovyeva & Pearce, 2011); however, the genetic variation and structure of the two breeding populations remain unclear. Last Glacial Maximum (LGM) and post‐LGM dynamics of landscapes have been suggested as a driver of range and genetic structure dynamics in several sea duck species (Pearce et al., 2009; Tiedemann et al., 2004). In contrast to glaciated territories of the Northern Europe and Arctic North America, the LGM and post‐LGM dynamics of climate and landscapes were not so dramatic in the South of Russian far East and in Northeast China Clime changes led to significant moving of the vegetation borders to the South (in LGM) and to the North (in mid‐Holocene and during climatic optimum), especially well pronounced between 38 and 54 N. Cooling during the LGM was accompanied by the reduction of yearly precipitation (Belyanin et al., 2019; Korotky et al., 2010; Zhao et al., 2003). Thus landscapes, preferred for the SSME breeding (Xu, Solovyeva, et al., 2021b), shifted southward during the LGM and shifted northward in mid‐Holocene. This may determine breeding range dynamics, and thus driving population differentiation for this riverine forest species. However, recent SSME population fluctuations have not been inferred while taking into account long‐term demographic progress.

In this study, genetic diversity and population structure of SSME breeding populations in the Sikhote‐Alin and Changbai Mountains were investigated, with an emphasis on evaluating the recent disturbances associated with the role of contemporary habitat loss and potential LGM‐related dynamics in the demographic history of the SSME breeding populations in Northeast China and Southeast Russia. With these analyses, the aims of this study were to help discern the historical paths and ongoing population processes in the SSME for conservation planning and to enrich our understanding of recent human‐induced demographic changes in endangered avian species.

## 2 METHODS

### 2.1 Mitochondrial DNA amplification and microsatellite genotyping

**FIGURE 1:**
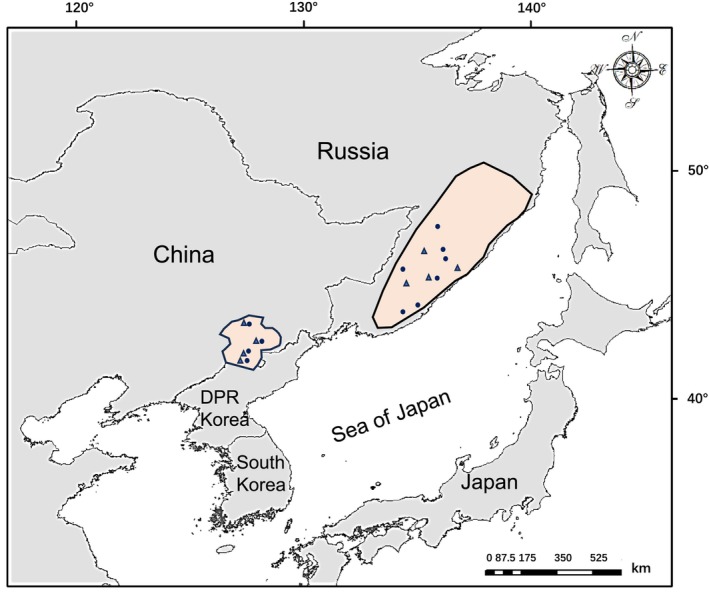
Distribution of main breeding areas (i.e., Changbai Mountains, China and Sikhote‐Alin Range, Russia; delineated by solid lines) of the scaly‐sided merganser (*Mergus squamatus*) and DNA sampling locations in East Asia (sites of mtDNA and microsatellites samples are represented by circles and triangles, respectively).

Mitochondrial DNA (mtDNA) sequences of control region from 59 individuals were amplified or downloaded for this study, including those obtained from noninvasive samples (nest lining feathers from 18 nests and membrane/dead embryo from three nests from 2019 to 2021) of breeding populations in Northeast China (CHN, *n* = 20) and Southeast Russia (RUS, *n* = 1) and from GenBank (accession numbers: HM639863–HM639866) for both breeding populations (RUS, *n* = 35; CHN, *n* = 3; Figure 1). DNA from the field samples was extracted using an animal tissue genomic DNA extraction kit (Sangon, Shanghai). A fragment of the control region (~ 405 bp) was obtained for the sampled individuals using primers described by Solovyeva and Pearce (2011), which were developed for European Goosanders (*Mergus merganser*; Hefti‐Gautschi et al., 2009). PCR reactions were performed in final volumes of 25 μL, containing approximately 1 μL of genomic DNA, 0.5 μL dNTPs (10 mmol/μL), 1 μL of each primer (10 mmol/μL), 2.5 μL Taq buffer (with MgCl_2_), and 0.2 μL Taq DNA polymerase (5 U/μL). The reactions were conducted with an initial denaturation step at 95°C for 5 min, followed by 38 cycles of denaturation at 94°C for 30 s, annealing at 58°C for 30 s, and extension at 72°C for 60 s. A final extension step at 72°C for 10 min was included. The haplotypes that differ from previously published sequences were deposited in GenBank under accession numbers PP828704‐PP828707.

Given that DNA obtained from noninvasive samples often contains limited genetic material, leading to low amplification success rates (Dai et al., 2015), we used a different batch of samples (lining feathers from 42 nests and membrane/dead embryo from four nests) to determine the motif nucleotide length for each individual. Between 2019 and 2021, a total of 21 samples were collected from RUS and 25 samples obtained from CHN (Figure 1). To identify polymorphic microsatellites (SSR), we initially tested loci from other genetically closely related species (Bouchard, 2002; Gautschi & Koller, 2005; Jaari et al., 2009; Maia et al., 2017). Genomic DNA was extracted using a Rapid Animal Genomic DNA Isolation Kit (Sangon, Shanghai). PCR amplification was performed in a total volume of 25 μL, which included 2.5 μL of 10 × PCR Buffer, 0.5 μL of a 10 mmol/μL dNTP mix, 2 μL of 25 mmol/μL MgCl_2_, 0.2 μL of 5 U/μL Taq polymerase, 0.5 μL of each primer, 17.8 μL of H_2_O, and approximately 50 ng of DNA. The thermal cycling conditions consisted of an initial denaturation at 95°C for 3 min, followed by 10 cycles of 95°C for 30 s, 60°C for 30 s, and 72°C for 30 s. This was followed by an additional 20 cycles of 95°C for 30 s, 55°C for 30 s, and 72°C for 30 s, with a final elongation step at 72°C for 6 min. The PCR products were analyzed by Songon Biotech in Shanghai, China, using an ABI PRISM 3730XL Genetic Analyzer. Polymorphism testing was conducted with GeneMapper v3.7 for size range determination. A total of nine polymorphic SSR loci were successfully amplified using primers developed for Goosander (Gautschi & Koller, 2005) and Barrow's goldeneye (*Bucephala islandica*; Jaari et al., 2009). In addition, we attempted to develop SSR loci through a next‐generation sequencing approach. Genomic DNA was isolated using a Rapid Animal Genomic DNA Isolation Kit (Sangon, Shanghai). Approximately 1 μg of DNA was sheared using a Covaris S220, and an Illumina paired‐end shotgun library was prepared following the standard protocol of the Illumina TruSeq DNA Library Kit. The libraries were pooled, and sequencing was performed on the Illumina HiSeq 2500 platform (paired‐end 125 bp). Over 2 million reads were analyzed with MISA 1.0 to extract sequences containing dimers, trimers, tetramers, pentamers, and hexamers. Primer design was conducted using Primer3 (Rozen & Skaletsky, 2000). Probably influenced by amount and quality of the material of noninvasive samples, which may contribute to low amplification efficiency to the loci with various repeat numbers (Cui et al., 2022; Dai et al., 2015), 15% of 50 candidate loci could be efficiently amplified specifically and only one locus was polymorphic (GeneBank accession number: PP860612). Finally, the 10 polymorphic SSR loci were selected, which can be powerful candidate markers for further evaluation and conservation of SSME genetic diversity.

### 2.2 Genetic diversity and differentiation analysis

We applied MEGA v6.0 software to align the sequences of the mtDNA fragments and used DnaSP v5.10 to estimate mtDNA diversity by calculating haplotype diversity (*h*) and nucleotide diversity (π) using 10,000 bootstrap replicates. The median joining of mitochondrial haplotypes was constructed in PopART using the TCS algorithm with a 95% connection limit (Leigh & Bryant, 2015). For SSR loci, we utilized Microchecker v2.2 to examine the null allele and allelic dropout and applied Cervus v3.0 to calculate the allele frequency, number of alleles (Na), expected heterozygosity (He), observed heterozygosity (Ho) and polymorphism information content (PIC).

Deviations from Hardy–Weinberg equilibrium and genotypic linkage disequilibrium (using Fisher's exact tests) were calculated with Genepop v4.7.5, with multiple comparisons adjusted using the Bonferroni correction. To assess genetic differences between the breeding populations of RUS and CHN, pairwise population Fst was calculated using GenAlEx v6.5 with 9999 permutations based on SSR data, and in Arlequin v3.01 with 10,000 permutations for mtDNA fragments. STRUCTURE v2.3.3 was employed to assign individuals to *K* = 2 clusters, performing 10 replicate runs for each *K* value. Monte Carlo Markov Chains (MCMCs) were run for 1,000,000 cycles using independent allele frequency models, with the first 20% of cycles discarded as burn‐in. Additionally, Principal Coordinate Analysis (PCoA) was performed in GenAlEx v6.5 using pairwise codominant genotypic genetic distances to visualize genetic similarities through multidimensional scaling. Gene flow was indirectly estimated using the values of Nm, calculated from Fst as Nm = (1 − Fst)/4Fst (Wright, 1978). The inbreeding coefficient (Fis) was calculated using Genepop v4.7.5.

### 2.3 Demographic inferences

The bottleneck‐induced genetic variability pattern, that is, higher‐than‐expected heterozygosity (Luikart et al., 2008), was evaluated in two mutational models (a stepwise mutational model, SMM; and a two‐phased stepwise mutational model, TPM) by assuming equilibrium of mutation drift. The Wilcoxon signed‐rank test was used for significance measurement because it reportedly has great statistical power when dealing with a small set of SSR loci and limited sample size (Cornuet & Luikart, 1996). In addition, evaluations based on mode shift were used to verify whether there was an absence of an L‐shaped allele frequency distribution pattern (which is a typical feature in stable populations; Luikart et al., 1998). To assess the history of expansion after the bottleneck, Fu's Fs and Tajima's D were estimated in Arlequin v3.01 using mtDNA fragments (Fu, 1997; Tajima, 1989). Based on the SSR dataset, we applied an approximate Bayesian computation (ABC) approach in DIYABC to discriminate the relative importance of LGM and post‐LGM dynamics and recent habitat destruction events on the present‐day pattern of population differentiation (Cornuet et al., 2014). The priors of population sizes and timing of the demographic changes are detailed in Table 3. The possibility of population divergence was compared among the three scenarios (S1–S3) by assuming the effects of LGM, post‐LGM colonization and habitat destruction during the 20th century. Based on the estimated optimal quantity of replicates, a total of 3,000,000 simulations of the scenarios were performed. Effective population size was assessed by the posterior estimation of demographic parameters from the ABC analysis. Finally, the demographic disequilibrium of the population was assessed by calculating the ratio *N*e/*N*, where *N* represents the census adult population size. Recently reduced populations generally display a *N*e/*N* <1. For the estimation of *N*, we used the census values during 2000–2012 (Solovyeva et al., 2014).

## 3 RESULTS

**FIGURE 2:**
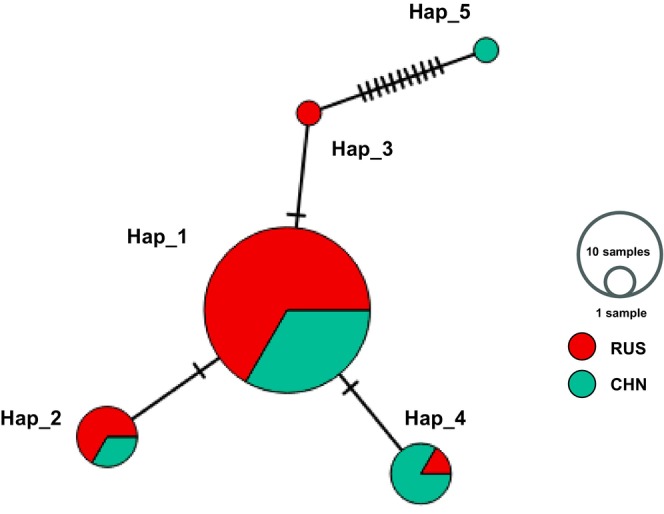
Median haplotype networks based on mtDNA control region sequences of samples collected from Southeast Russia (RUS) and Northeast China (CHN). The circle size is proportional to the number of individuals, and the fractions are proportional to the frequencies of samples per haplotype.

Five mtDNA haplotypes were identified among the sequences of fragments in the control region. There were three haplotypes null shared by both the RUS and CHN populations, with inconsistent haplotype frequency distributions between the two areas (Table S1; Figure 2). In addition, two singleton haplotypes were observed in both the RUS and CHN samples (Table S1; Figure 2). Although the number of haplotypes was the same between the two local populations, the haplotype diversity and nucleotide diversity were different between CHN (*h* = 0.542; *π* = 0.00388) and RUS (*h* = 0.300; *π* = 0.00078). The genetic diversity of mtDNA in the SSME was greater than that in the critically endangered Brazilian merganser but was markedly less than that in several other mergansers (Table 1).

**TABLE 1:** Comparison of population genetic parameters inferred from mitochondrial DNA control region sequence from the scaly‐sided merganser and other three species of merganser.

Species	*N*	Haplotypes (H)	Haplotype diversity (*h*)	Nucleotide diversity (*π*)	References
Scaly‐sided merganser (EN)	59	5	0.404	0.00078	This study
Scaly‐sided merganser	38	4	0.292	0.00070	Solovyeva and Pearce (2011)
Common merganser	24	6	0.734	0.00380	Solovyeva and Pearce (2011)
Common merganser	203	42	0.918	0.01300	Hefti‐Gautschi et al. (2009)
Common merganser	130	32	\	\	Pearce et al. (2009)
Red‐breast merganser	20	9	0.833	0.00550	Solovyeva and Pearce (2011)
Red‐breast merganser	64	25	\	\	Pearce et al. (2009)
Brazilian merganser (CR)	56	1	0	0	Maia et al. (2020)

*Note*: EN and CR represents for endangered species and critically endangered, respectively.

**FIGURE 3:**
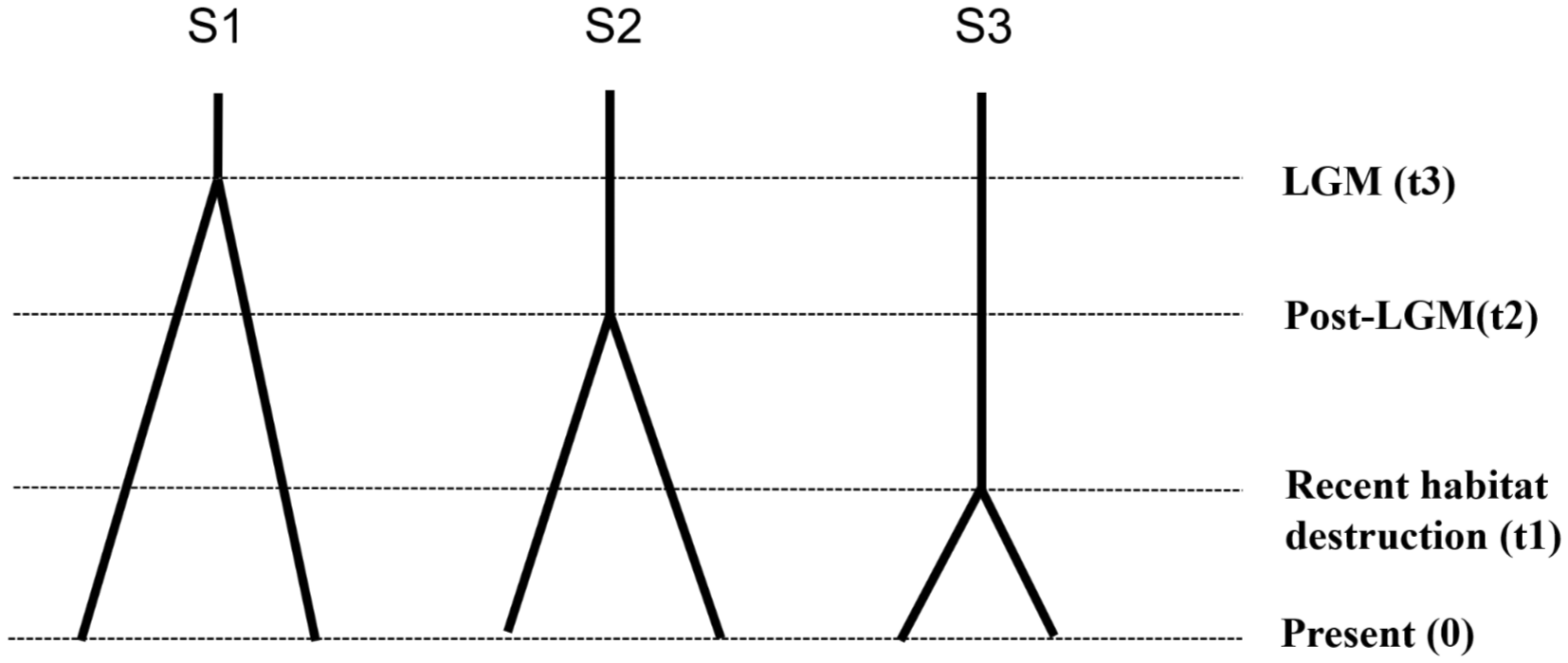
Approximate Bayesian computation (ABC) scenarios representing the demographic history of the scaly‐sided mergansers (*Mergus squamatus*) from the past (upper part) to present (lower part). S1 ~ S3 represents population divergence during Last Glacial Maximum (LGM), post‐LGM and recent period of habitat destruction.

**FIGURE 4:**
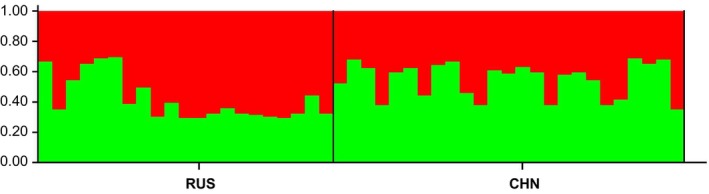
Bayesian STRUCTURE based on 10 microsatellites of breeding population of the scaly‐sided mergansers (*Mergus squamatus*) at Southeast Russia (RUS) and Northeast China (CHN).

**FIGURE 5:**
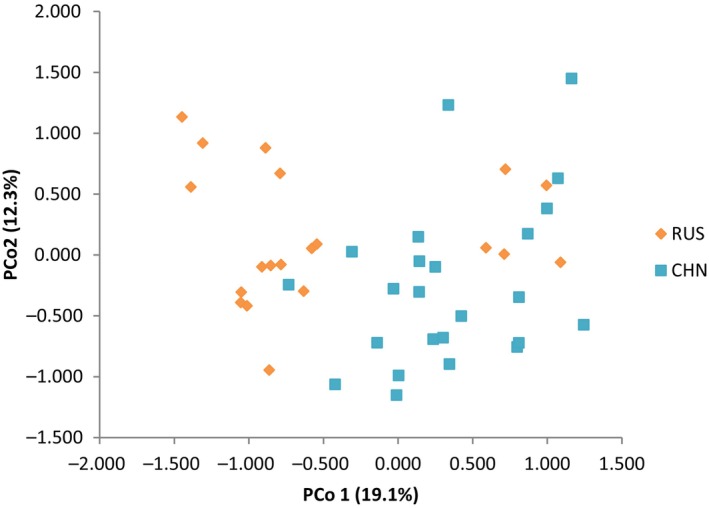
Principal coordinates analysis (PCoA) based on microsatellite genotypes for the 46 scaly‐sided mergansers (*Mergus squamatus*) of Southeast Russia (RUS) and Northeast China (CHN).

There were no null alleles or large allele drop outs at the SSR loci of Bisl10, Bisl15, Bisl21, MM01, MM05, MM06, or SSME91 in RUS and CHN. A general excess of homozygotes for most allele size classes was observed at the Bisl16 locus in RUS and at the Bisl20 locus in CHN. A significant departure from Hardy–Weinberg equilibrium, after applying the sequential Bonferroni correction (Rice, 1989), was detected at the Bisl16 locus in RUS and the Bisl21 locus in CHN. No significant linkage disequilibrium was found between any pair of loci. Given that the presence of null alleles and loci deviating from Hardy–Weinberg equilibrium can potentially bias population genetic analyses, SSR‐based analyses were also conducted excluding the three loci (Bisl16, Bisl20, and Bisl21). Since the results remained consistent with those obtained using the complete set of loci (as detailed in Appendix S1), these alleles were included in the subsequent analyses. All 10 loci were polymorphic with a mean PIC >0.5 (0.639; ranging from 0.350 to 0.782), indicating high polymorphism among the different loci in both RUS and CHN (Table 2). The mean number of alleles (6.4 and 6.1) and average observed heterozygosity (0.719 and 0.689) at SSR loci in RUS and CHN were greater than those found in other merganser species (Hefti‐Gautschi et al., 2009; Maia et al., 2020). Pairwise population differentiation analysis revealed weak but significant population differentiation, inferred from mtDNA fragments (Fst = 0.035, *p* = .048) and overall SSR loci (Fst = 0.032, *p* = .001). According to Wright (1978), there was a high level of gene flow (Nm = 7.56) between the two local populations. The inbreeding coefficient (Fis) of the breeding populations in RUS and CHN was estimated at −3.10% (*p* = 1.00). Principal Component Analysis (PCA) confirmed the STRUCTURE results (*K* = 2), indicating that the two local populations tend to separate. (Figure 4; Figure 5). Under the assumptions of the SMM (probability of H excess = 0.98779 and 0.88379) and TPM (probability of H excess = 0.31250 and 0.21582), no evidence of significant deviation from mutation‐drift equilibrium was found. Consistently, the “mode‐shift” indicator did not deviate from the regular L‐shaped pattern, suggesting the presence of a constant population without recent genetic bottlenecks (Luikart et al., 1998). Although the significant negative values of two demographic parameters (RUS Tajima's D = −2.07812, *p* = .00400; and CHN Fu's Fs = −2.14055, *p* = .027**)** suggested historical expansion, the results of the other two estimators (RUS Fu's Fs = 1.28203, *p* = .7840; CHN Tajima's D = −1.26655, *p* = 0.09200) were inconsistent. Simulations based on the ABC approach revealed that S3 had the significantly highest posterior possibility (>0.9999) when compared with the other two scenarios assuming LGM and post‐LGM demographic dynamics (Figure 3). The population was estimated to have differentiated approximately 66 ya ago (Table 3), representing the most severe habitat destruction period (1950s ~ 2000s) induced by anthropogenic effects in the breeding grounds of Russia and China (see Discussion). The posterior estimation of demographic parameters from Approximate Bayesian Computation (ABC) analysis indicated an effective population size (Ne) of 1570 individuals (95% CI: 1080–3760) in RUS and 380 individuals (95% CI: 137–393) in CHN. Considering the reported numbers of breeding pairs from recent census data (approximately 1640 pairs in RUS and 155 pairs in CHN), the ratio of effective population size to census breeding population size (Ne/N) was 0.48 for RUS and 1.23 for CHN.

**TABLE 2:** Summary statistics from 10 microsatellites loci used for scaly‐sided mergansers (*Mergus squamatus*) from Southeast Russia (RUS) and Northeast China (CHN).

Pop	Locus	*N*	Na	Ho	He	PIC
RUS	Bisl10a	21	7.000	0.905	0.766	0.735
	Bisl11a	21	8.000	0.905	0.768	0.738
	Bisl15a	21	4.000	0.714	0.636	0.564
	Bisl16a	21	5.000	0.238	0.567	0.510
	Bisl20a	21	8.000	0.857	0.795	0.768
	Bisl21a	21	7.000	0.952	0.756	0.723
	SSME91a	21	3.000	0.286	0.421	0.350
	MM01a	21	9.000	0.714	0.746	0.724
	MM05a	21	6.000	0.762	0.666	0.621
	MM06a	21	7.000	0.857	0.803	0.772
	Mean	21	6.400	0.719	0.692	0.651
CHN	Bisl10a	25	7.000	0.680	0.729	0.687
	Bisl11a	25	7.000	0.920	0.803	0.775
	Bisl15a	25	3.000	0.600	0.550	0.463
	Bisl16a	25	5.000	0.640	0.566	0.490
	Bisl20a	23	7.000	0.565	0.771	0.738
	Bisl21a	25	6.000	1.000	0.701	0.649
	SSME91a	25	4.000	0.440	0.450	0.392
	MM01a	24	9.000	0.750	0.823	0.800
	MM05a	24	6.000	0.375	0.543	0.490
	MM06a	25	7.000	0.920	0.808	0.782
	Mean	24.6	6.100	0.689	0.674	0.627

**TABLE 3:** Prior distribution and posterior estimation of demographic parameters of the breeding populations of the scaly‐sided mergansers (*Mergus squamatus*) using the best fit scenario S3 of the approximate Bayesian computation (ABC) analysis.

Priors			Posterior estimates of scenario 3		
Parameters	Min	Max	Mode	q0.05	q0.95
N1	10	4000	1570	1080	3760
N2	10	400	380	137	393
*t*3	15,000	100,000	\	\	\
*t*2	1000	20,000	\	\	\
*t*1	10	1000	66	30	207

*Note*: N1 and N2 are the present‐day effective population sizes of the Scaly‐sided merganser at Southeast Russia and Northeast China. *t*x corresponds to time priors, expressed in years: *t*1, beginning of the habitat destruction; *t*2, beginning of the colonization during post‐glaciation; *t*3, beginning of last glaciation. q0.05 and q0.95 are estimates of the respective confidence limits.

## 4 DISCUSSION

In accordance with comparative mitochondrial genetic studies of mergansers (Solovyeva & Pearce, 2011), which have focused mainly on Russian populations, the SSME breeding populations of both Russia and China were shown to have limited mtDNA diversity in the control region. Low mtDNA genetic diversity can be shaped by various historical and demographic processes. Corresponding with the contemporary dynamics of breeding populations (Solovyeva et al., 2014), historical expansion after a bottleneck was suggested by estimates of Tajima's D in RUS and Fu's Fs in CHN (Solovyeva & Pearce, 2011). However, the Fu's Fs and Tajima's D in RUS and CHN, respectively, provided inconsistent evidence, demonstrating that there was a constant historical population with no large fluctuations in effective population size. Although the Ne/N ratio suggests a population reduction in RUS, which aligns with evidence of breeding population declines in their distribution regions since the mid‐20th century (BirdLife International, 2001), we found no genetic signatures of recent demographic bottlenecks in either the RUS or CHN populations. It is possible that bottleneck effects resulting from populations experiencing recent reductions are undetectable (Peery et al., 2012). A few factors, such as the intensity of population decreases, duration of population declines and expansion, population size, and genetic variability before population declines, can interfere with tests of bottlenecks (Cornuet & Luikart, 1996; Funk et al., 2010; Luikart et al., 1998; Williamson‐Natesan, 2005). Population declines could be effectively detected when the proportion of reductions is sufficiently high or when the population rapidly becomes very small (Peery et al., 2012). The absence of a bottleneck signal probably resulted from limited demographic changes and/or the original lack of genetic variation.

It is likely that the limited historical population and narrow range of breeding habitats constrained the genetic variability. Breeding SSME populations are confined to submountain rivers with clear water, wide, deep and sinuous channels, gravel/rocky substrates with large gravel bars and riparian old growth forests (IUCN, 2017; Xu, Wang, et al., 2021a), the amounts of which are small within the already restricted distribution range (Xu, Solovyeva, et al., 2021b). In particular, female SSMEs are among the largest cavity‐nesting bird species and are confined by the limited large cavities of older trees, as there may otherwise be a cost of looking for suitable cavities in unfamiliar habitats. Coinciding with these constraints, the total effective population was estimated to be small (1570 and 380 individuals in RUS and CHN; Table 3), which is approximately half of the surveyed population size (i.e., appr. 2000 pairs; Solovyeva et al., 2014). As genetic diversity is expected to be proportional to effective population size (Ellegren & Galtier, 2016), the potentially limited historical population in a narrow habitat range probably prevents SSMEs from obtaining higher mtDNA diversity.

In contrast to those inferred from mtDNA, the level of genetic diversity inferred from microsatellite loci was greater than that inferred from other merganser species (Hefti‐Gautschi et al., 2009; Maia et al., 2020). Given the potential for females to mate with males from different breeding sites at wintering grounds, the genetic diversity of nuclear DNA may not be as restricted as that of mtDNA and may be mediated by male dispersal (Hefti‐Gautschi et al., 2009). The high level of gene flow may be an important mechanism for maintaining intraspecific genetic diversity (Slatkin, 1994). In addition, hybridization and introgression with related sympatric species, for instance, Goosanders (Surmach & Zaykin, 1994), could also contribute to the maintenance of high levels of SSR genetic diversity (Mallet, 2005; Peters et al., 2005), which deserves further investigation.

Even though genetic differentiation could be expected when considering the discrete clustering distribution and potential female philopatry, the overall population structure was very weak, which is inconsistent with the spatial genetic patterns of other least concerning or critically endangered sea duck species (Brown et al., 2020; Hefti‐Gautschi et al., 2009; Maia et al., 2020; Pearce et al., 2005, 2009; Scribner et al., 2001). The SSME is among the largest cavity‐nesting waterfowl species, which may limit it to a smaller proportion of natural cavities. The cost of dispersal, in terms of locating large cavities in unfamiliar habitats, likely encourages females to return to their breeding grounds. Consistently, geolocator data reveal that SSME exhibit fidelity to nesting areas (Solovyeva et al., 2012). While strong female population structure may be maintained by philopatry, high levels of winter dispersal among closely nesting females are suggested as a potential mechanism for maintaining gene flow in this threatened species (Solovyeva et al., 2012), which may contribute to the observed weak population structure. In addition, it is important to consider various demographic and historical factors when explaining the low level of genetic divergence in the SSME. For migratory avian species, there is a range of phylogeographic structures owing to demographic processes associated with the degree of late Pleistocene fragmentation and recolonization in the post‐LGM period (Jones et al., 2005; Mille et al., 2023). In contrast, species that lack strong genetic structure are thought to have evolved from a single ancestral refuge (Wojczulanis‐Jakubas et al., 2014). By rejecting the probability of divergence during LGM and post‐LGM periods, the DIYABC results support the latter scenario in the SSME. Additionally, considering the historical consecutive distribution of SSME, which is primarily concentrated within a narrow geographical range of Northeast Asia (BirdLife International, 2001), the breeding populations may have originated from a single homogeneous refugia population.

Although potential male‐biased dispersal could undermine the impact of genetic drift and homogenize populations (Slatkin, 1989), leading to a lack of genetic structure among the populations, significant genetic differentiation was inferred from both female inherited sequences and biparentally transmitted microsatellites. The weak overall genomic differentiation between the local populations can be explained by recent divergence (e.g., Clark et al., 2022). Like several other studies of endangered species (Tracy & Jamieson, 2011; Wang et al., 2019), DIYABC analysis revealed contemporary but not historical genomic differentiation between the breeding populations (i.e., S3). In addition, the estimated divergence time (approximately the 1950s) coincided with the period of habitat destruction and population declines in the breeding ground. In accordance with these estimations, the SSME breeding population is believed to have contracted to the current two mountainous areas only recently (Solovyeva et al., 2014). With social and economic developments from the 1950s to the 2000s in China, forest logging, hydroelectric construction, and other human disturbances, tremendous habitat alterations have taken place in northeastern China, likely excluding this species from large areas of the historical breeding region (BirdLife International, 2001; Liu et al., 2010). During the intense economic development of the 1960s–1970s in Russia, primary forests along almost all large submountain rivers were greatly altered, with SSME numbers rapidly declining in the northern Sikhote‐Alin Mountains (IUCN, 2017). Following recent changes in breeding habitats, it may be difficult for recently isolated populations to reach equilibrium among mutation, migration, and genetic drift (Whitlock & McCauley, 1999). Given the recent distribution dynamics of the breeding SSME (Liu et al., 2010; Solovyeva et al., 2014), the populations are supposed to be in a nonequilibrium state, which may contribute to the shallow population structure (Friesen et al., 1996; Moum & Arnason, 2001; Wojczulanis‐Jakubas et al., 2014).

### 4.1 Conservation implications

A depletion in the genetic diversity of wild populations is related to reduced reproduction and increased susceptibility to infectious diseases (Reed & Frankham, 2003). These challenges pose a threat to the reproductive success and long‐term survival of populations (Frankham, 2005). Despite the high level of nuclear genetic diversity, low maternal inherited mtDNA diversity could result in sex‐biased fitness effects (Smith & Connallon, 2017). SSMEs had a small population size and a smaller estimated effective population, with the population more restricted in CHN (which inhabited only 1/10 of the breeding population and 1/5 of the estimated effective population) than in RUS. Despite the low level of genetic structure, unique haplotypes, and different genotypic diversities were exhibited for the RUS and CHN populations. If breeding populations in CHN suffered from a further severe population decline, it is possible that they would not recover in a short period through the recruitment of females from other habitats. Given that the CHN population is not completely exchangeable with the larger RUS population, we suggest conserving the small breeding habitat independently. In addition, since the population in RUS may have experienced a more drastic decline (Ne/N < 1) compared to that in CHN (Ne/N > 1), RUS should be considered a priority area for conservation efforts. In consequence, conservation efforts should be strengthened for SSMEs in both Russia and China. To mitigate the extinction threats imposed by recent genetic isolation, the recovery of breeding habitats and the restoration of connectivity between habitat fragments are assumed to be priorities in conservation.

## 5 CONCLUSION

To our knowledge, this study is the first to assess the genetic diversity, population structure, and demographic history of endangered SSME populations by using both mtDNA sequence information and SSR loci. Analysis of the different molecular markers suggested limited female‐inherited genetic diversity and a high level of nuclear genetic diversity. In addition, analysis of both markers consistently revealed significant but weak divergence between the breeding populations. In demographic history analysis, although a stable historical effective population size without drastic bottlenecks cannot be rejected, signal of population decline was detected in RUS. In addition, the RUS and CHN populations were suggested to have started to genetically diverge from each other due to the anthropogenic effects of recent fragmentation events. These results suggest that small historical population size and shallow evolutionary history may be potential factors contributing to the contemporary genetic diversity pattern of breeding SSME. Conservation efforts should focus on protecting current breeding habitats from further destruction (loss and fragmentation) and restoring connected suitable breeding grounds among RUS and CHN.

## AUTHOR CONTRIBUTIONS

**Yulong Shen:** Data curation (equal); formal analysis (lead); methodology (lead); software (equal); writing – original draft (equal). **Ye Gong:** Conceptualization (lead); data curation (equal); funding acquisition (lead); project administration (lead); resources (equal); supervision (lead); validation (lead); visualization (lead); writing – original draft (equal); writing – review and editing (lead). **Diana Solovyeva:** Conceptualization (supporting); resources (equal); validation (equal). **Lin Wang:** Methodology (equal); resources (equal); software (equal). **Mu Li:** Formal analysis (equal); investigation (equal). **Mengxuan Hu:** Investigation (equal). **Yiwei Jiang:** Investigation (equal). **Sergey Vartanyan:** Resources (equal); writing – review and editing (equal). **Haitao Wang:** Project administration (equal); supervision (equal).

## CONFLICT OF INTEREST STATEMENT

The authors declare no conflicts of interest.

## Supporting information


Appendix S1.

## Data Availability

The datasets of the microsatellite loci analyzed during the current study are available in the Zenodo repository at DOI: 10.5281/zenodo.10574351.
