# Supplementary material for: Genetic diversity and shallow genetic differentiation of the endangered scaly‐sided merganser Mergus squamatus
Source: Ecol Evol. 2024 Jul 9;14(7):e70011. doi: 10.1002/ece3.70011 (PMC11233196; doi:10.1002/ece3.70011)
Supplement: Supplementary file 1 — Appendix S1. [file ECE3-14-e70011-s001.docx]

**Table S1** Mitochondrial DNA haplotype (H), location of variable sites, and numbers of each haplotype observed among scaly-sided mergansers in Southeast Russia (RUS) and Northeast China (CHN).

| H | variable sites | | | | | | | | | | | | | | Location | | Total | |
| --- | --- | --- | --- | --- | --- | --- | --- | --- | --- | --- | --- | --- | --- | --- | --- | --- | --- | --- |
|  |  |  |  |  |  |  |  |  |  |  |  |  |  |  | RUS | CHN |  | |
|  | 7 | 33 | 34 | 35 | 82 | 84 | 85 | 89 | 122 | 138 | 141 | 161 | 174 | 197 |  |  |  |  |
| #1 | t | c | t | t | t | a | c | t | t | c | t | a | g | t | 15 | 30 | 45 |  |
| #2 | t | c | t | t | t | a | t | t | t | c | t | a | g | t | 2 | 4 | 6 |  |
| #3 | t | c | t | t | t | a | c | t | t | c | t | a | g | t | 5 | 1 | 6 |  |
| #4 | t | c | t | t | t | a | c | t | c | c | t | g | g | t | 0 | 1 | 1 |  |
| #5 | a | t | c | c | c | g | c | c | t | t | c | g | a | c | 1 | 0 | 1 |  |

**Table S2** Information of microsatellite loci

| **Locus** | **size （bp）** | **Repeat motif** | **Na** | **Source** |
| --- | --- | --- | --- | --- |
| MM01 | 138-152_（14）_ | （CA）_17_ | 7 | *Mergus merganser* |
| MM05 | 178-206_(28)_ | （AC）_23_ | 8 | *Mergus merganser* |
| MM06 | 103-117_(14)_ | （CA）_22_ | 3 | *Mergus merganser* |
| Bisl10 | 142-182_(40)_ | （TCGA）_16_ | 5 | *Bucephala islandica* |
| Bisl11 | 203-231_(28)_ | （TCTA）_15_ | 7 | *Bucephala islandica* |
| Bisl15 | 270-298_(28)_ | （TATC）_11_ | 7 | *Bucephala islandica* |
| Bisl16 | 173-207_(34)_ | （ATAG）_11_ | 4 | *Bucephala islandica* |
| Bisl20 | 209-235_(26)_ | （TC）_19_ | 10 | *Bucephala islandica* |
| Bisl21 | 213-242_(29)_ | （TTCT）_15_ | 6 | *Bucephala islandica* |
| SSME91 | 172-178_(6)_ | （TG）_12_ | 8 | PP860612^a^ |

^a^ GeneBank accession number of the SSR locus developed through a next-generation sequencing approach

By excluding the three loci that with null alleles or deviating from Hardy–Weinberg equilibrium, the results of genetic population analyses remain consistent. Pairwise population differentiation analysis revealed weak but significant population differentiation, inferred from the SSR loci (Fst = 0.025, p = 0.001). According to Wright (1978), there was a high level of gene flow (Nm = 9.75) between the two local populations. Inbreeding coefficient (Fis) of the breeding SSME of RUS and CHN was estimated as -3.40%, which was nonsignificant (p = 0.99). The principal component analysis (PCA) analysis and the structure results (K =2) suggested that the two local populations tend to separate (Fig. S4; Fig. S5). Under the assumptions of the SMM (probability of H excess = 0.96 and 0.77) and TPM (probability of H excess = 0.47 and 0.23), no evidence of significant deviation from mutation-drift equilibrium was found. Consistently, the ‘‘mode-shift’’ indicator did not deviate from the regular L-shaped pattern, suggesting the presence of a constant population without recent genetic bottlenecks (Luikart et al. 1998). Simulations based on the ABC approach revealed that S3 had the significantly highest posterior possibility (>0.9999) when compared with the other two scenarios assuming glacial and postglacial demographic dynamics. The population was estimated to have differentiated approximately 49.5 ya ago (Table S3), representing the most severe habitat destruction period (1950s~2000s) induced by anthropogenic effects in the breeding grounds of Russia and China. The posterior estimation of demographic parameters of ABC analysis showed Ne=1990 individuals (95% CI: 884; 3750) in RUS and Ne=395 individuals (95% CI) 112; 391) in CHN. By considering the reported number of breeding population in recent census data (approximate 1640 and 155 breeding pairs). The ratio of effective population size to census breeding population size (Ne/N) was 0.61 and 1.27 respectively.

**Table S3** Prior distribution and posterior estimation of demographic parameters of SSME using the best fit scenario S3

| **Piors** |  |  | | **Posterior estimates of scenario 3** | | |
| --- | --- | --- | --- | --- | --- | --- |
| **Parameters** | **Min** | **Max** | **Mode** | | **q0.05** | **q0.95** |
| N1 | 10 | 4000 | 1990 | | 884 | 3750 |
| N2 | 10 | 400 | 395 | | 112 | 391 |
| t3 | 15000 | 100000 | \ | | \ | \ |
| t2 | 1000 | 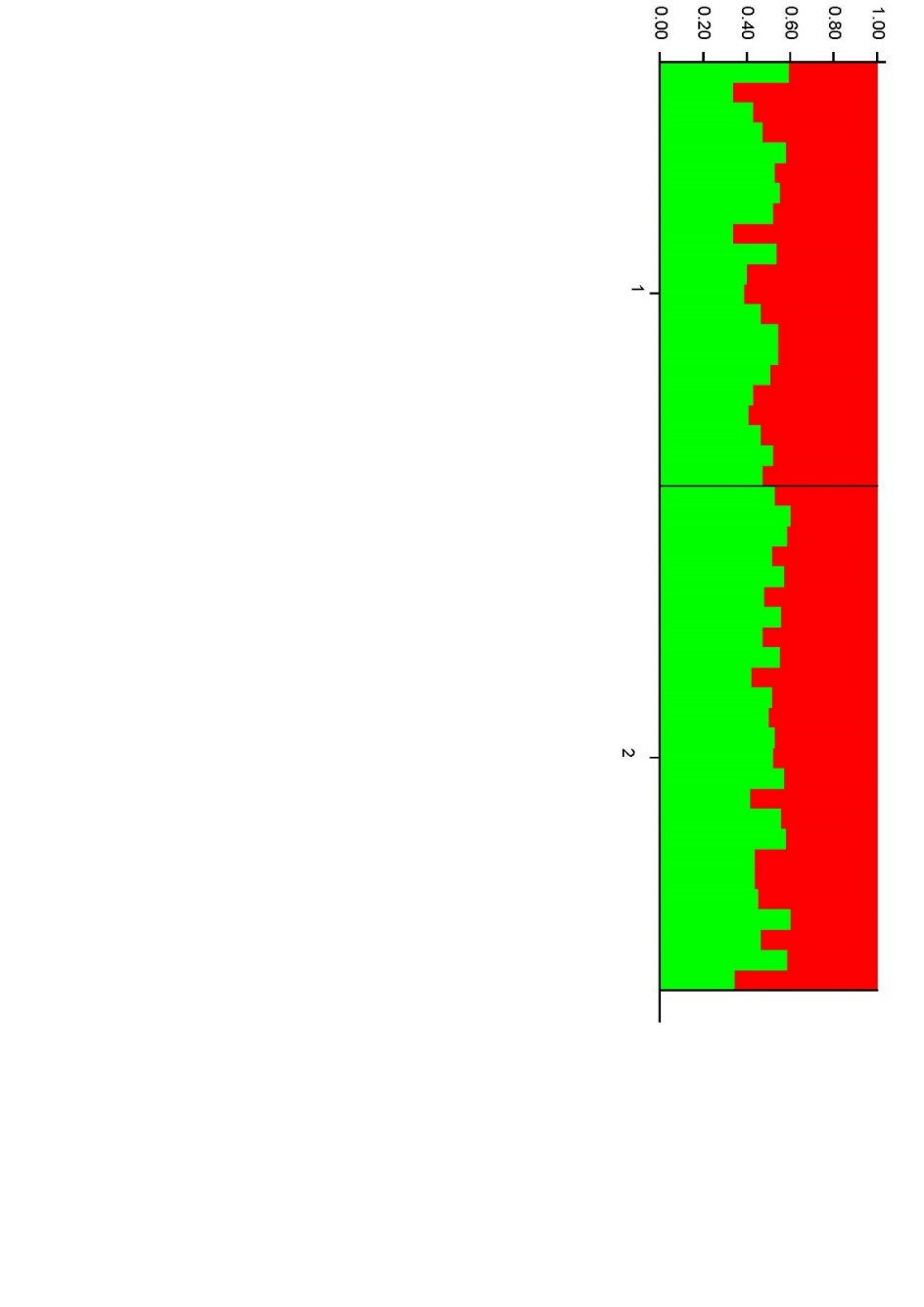20000 | \ | | \ | \ |
| t1 | 10 | 1000 | 49.50 | | 23.04 | 201.90 |

*Notes:* N1 and N2 are the present-day effective population sizes of *Mergus squamatus* at Southeast Russia and northeast China. *tx* corresponds to time priors, expressed in years: *t*1, beginning of the habitat destruction; *t*2, beginning of the colonization during post-glaciation; *t*3, beginning of last glaciation. q0.05 and q0.95 are estimates of the respective confidence limits.

**Figure S1** Bayesian STRUCTURE based on seven microsatellites of breeding population of the Scaly sided merganser at Southeast Russia (=1) and Northeast China (=2).

**Figure S2** Principal coordinates analysis (PCoA) based on microsatellite genotypes for the 46 Scaly sided mergansers of Southeast Russia (RUS) and Northeast China (CHN).
